# A protocol for ultra-high field laminar fMRI in the human brain

**DOI:** 10.1016/j.xpro.2021.100415

**Published:** 2021-03-26

**Authors:** Ke Jia, Elisa Zamboni, Catarina Rua, Nuno Reis Goncalves, Valentin Kemper, Adrian Ka Tsun Ng, Christopher T. Rodgers, Guy Williams, Rainer Goebel, Zoe Kourtzi

**Affiliations:** 1 Department of Psychology, University of Cambridge, Cambridge CB2 3EB, UK; 2 Wolfson Brain Imaging Centre, Department of Clinical Neurosciences, University of Cambridge, Cambridge CB2 0QQ, UK; 3 Department of Cognitive Neuroscience, Faculty of Psychology and Neuroscience, Maastricht University, Maastricht 6200 MD, the Netherlands; 4 Department of Industrial and Manufacturing Systems Engineering, The University of Hong Kong, Hong Kong SAR, China

**Keywords:** Clinical Protocol, Neuroscience, NMR

## Abstract

Ultra-high field (UHF) neuroimaging affords the sub-millimeter resolution that allows researchers to interrogate brain computations at a finer scale than that afforded by standard fMRI techniques. Here, we present a step-by-step protocol for using UHF imaging (Siemens Terra 7T scanner) to measure activity in the human brain. We outline how to preprocess the data using a pipeline that combines tools from SPM, FreeSurfer, ITK-SNAP, and BrainVoyager and correct for vasculature-related confounders to improve the spatial accuracy of the fMRI signal.

For complete details on the use and execution of this protocol, please refer to Jia et al. (2020) and Zamboni et al. (2020).

## Highlights

• Protocol for using ultra-high field imaging to measure activity in the human brain
• Defining cortical layers using high-resolution anatomical scans
• Preprocessing functional brain imaging data across cortical depth
• Correcting for vasculature-related confounds to improve spatial specificity

## Graphical abstract

**Figure:**
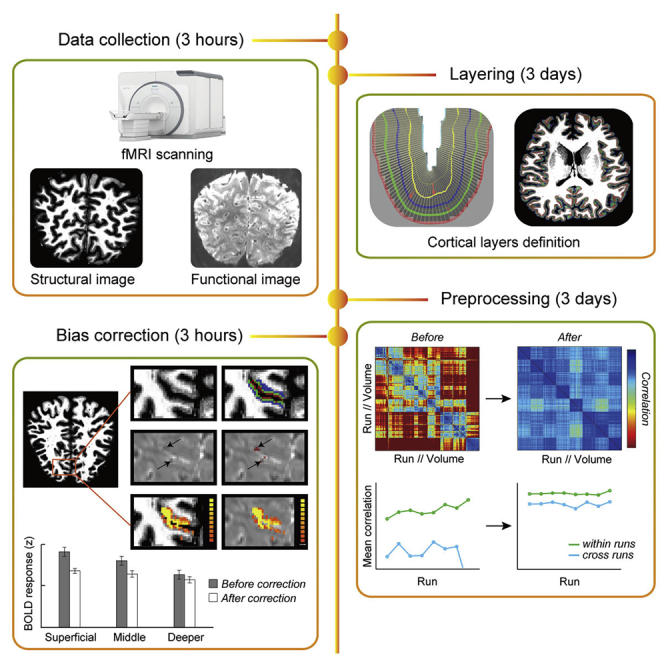

## Before you begin

### Participant screening

**Timing: 1 h per participant (1 day before scanning)**

1. Participants complete a safety screening form including exclusion criteria related to safety in the MR scanner (~10 min).
2. Participants complete the consent form to ensure that they understand the motivation of the study and any risks/benefits associated with it (~5 min). Then, we introduce our experiment to the participants (~5 min), give them instructions on what they need to do during the scan (~5 min), and ask them to practice the experimental task (~20 min).
3. Participants complete a personal information sheet, including questions related to health (age, medication, visual acuity etc., ~10 min).

### Scan setup for brain imaging at 7T (Siemens Terra scanner)

**Timing: 30 min per participant**

4. Turn on the stimulus computer and check the resolution and frame rate are set appropriately based on the experimental design.
5. Check whether scanner triggers and button box responses are registered by the stimulus computer.
6. Check the projection screen is placed in the correct position based on the viewing distance.
7. Ask the participant to change into MR appropriate scrubs and check for items that are not MR-compatible and not safe to bring into the scanner room. Ask the participant to lie on the scanner table in a comfortable position. Participants will require earplugs, pillows for the legs, immobilization cushions, respiratory belt, and a pulse oximeter. Note, this is a list of items / equipment necessary for visual experiments and additional items may be needed for other experiments. Remind participants not to cross their hands or legs. Explain to participants that they may squeeze the safety ball if they feel uncomfortable.

**CRITICAL:** Move the table into the scanner bore slowly to avoid the participants experiencing dizziness. If the participant feels dizzy, interrupt the table from moving into isocenter. This will allow the participant to recover from the rapid field change. Once the participant is happy to proceed, move the table towards isocenter.

**CRITICAL:** Ask the participant to stay as still as possible. Head movement may cause image distortions that cannot be corrected. Therefore, the researcher may communicate with participants using Yes or No questions shown on the screen. Ask the participants to answer these questions by pressing buttons. Remind the participant not to talk unless necessary.

**CRITICAL:** The Health and Safety Executive limit set for occupational exposure to sound is 85 dB without ear protection (see https://www.hse.gov.uk/noise/employers.htm for details). However, most sequences used in the high field scanner are very loud (around 110 dB). Hence all participants being scanned and those remaining inside the magnet room require ear protection. Earplugs offer up to 37 dB of attenuation with additional attenuation offered by the dense immobilization cushions. The researcher should carefully instruct the participants on the proper use of earplugs, and verify good fit and function of hearing protection in place prior to starting the scan.

**Figure 1:**
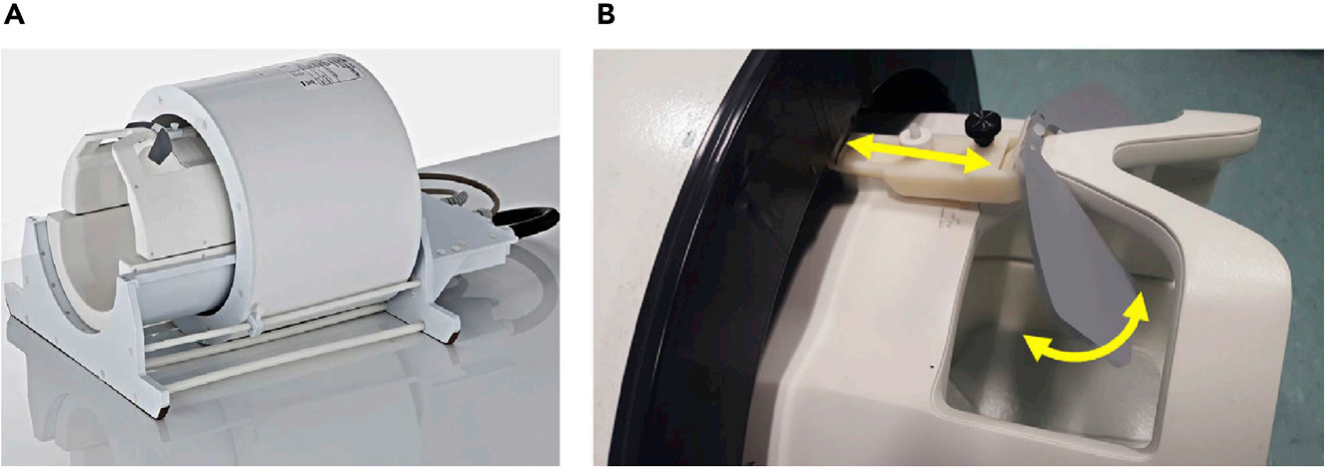
NOVA head coil and the mirror system (A) and (B) show images of the NOVA head coil and the mirror system, respectively.

8. Adjust the mirror system to check the visible area of the screen before the participant has moved into the scanner bore. Field of view may be limited when using the 32-channel phased array head coil (Nova Medical, Inc., Wilmington, MA, USA, Figure 1) for brain imaging on 7T scanners. If you notice that the participant cannot see enough of the screen, ask them to adjust their head so that they can see as much of the screen as possible, yet ensuring that also the participant’s head is placed the furthest back possible inside the head coil.

**CRITICAL:** If stimuli are not fully visible on the screen, you may need to display the stimuli at a different location by adapting the stimulus presentation scripts.

## Key resources table

**Table:** 

REAGENT or RESOURCE	SOURCE	IDENTIFIER
**Software and algorithms**		

MATLAB	https://www.mathworks.com/products/matlab.html	RRID: SCR_001622
Psychtoolbox	http://psychtoolbox.org/	RRID: SCR_002881
FreeSurfer	http://surfer.nmr.mgh.harvard.edu/	RRID: SCR_001847
ITK-SNAP	http://www.itksnap.org/	RRID: SCR_002010
BrainVoyager	http://www.brainvoyager.com/	RRID: SCR_013057
SPM	http://www.fil.ion.ucl.ac.uk/spm/	RRID: SCR_007037
mcheck toolbox	N/A	https://doi.org/10.17863/CAM.60330
MP2RAGE/EPI sequences	N/A	https://doi.org/10.17863/CAM.60568

**Experimental models: organisms/strains**		

Human subjects	Community of University of Cambridge (age range: 18–35, both genders)	N/A

**Other**		

Siemens Terra scanner	Siemens Healthineers	N/A

## Step-by-step method details

### Collecting structural and functional imaging data

**Timing: Approx. 120 min per participant**

**Figure 2:**
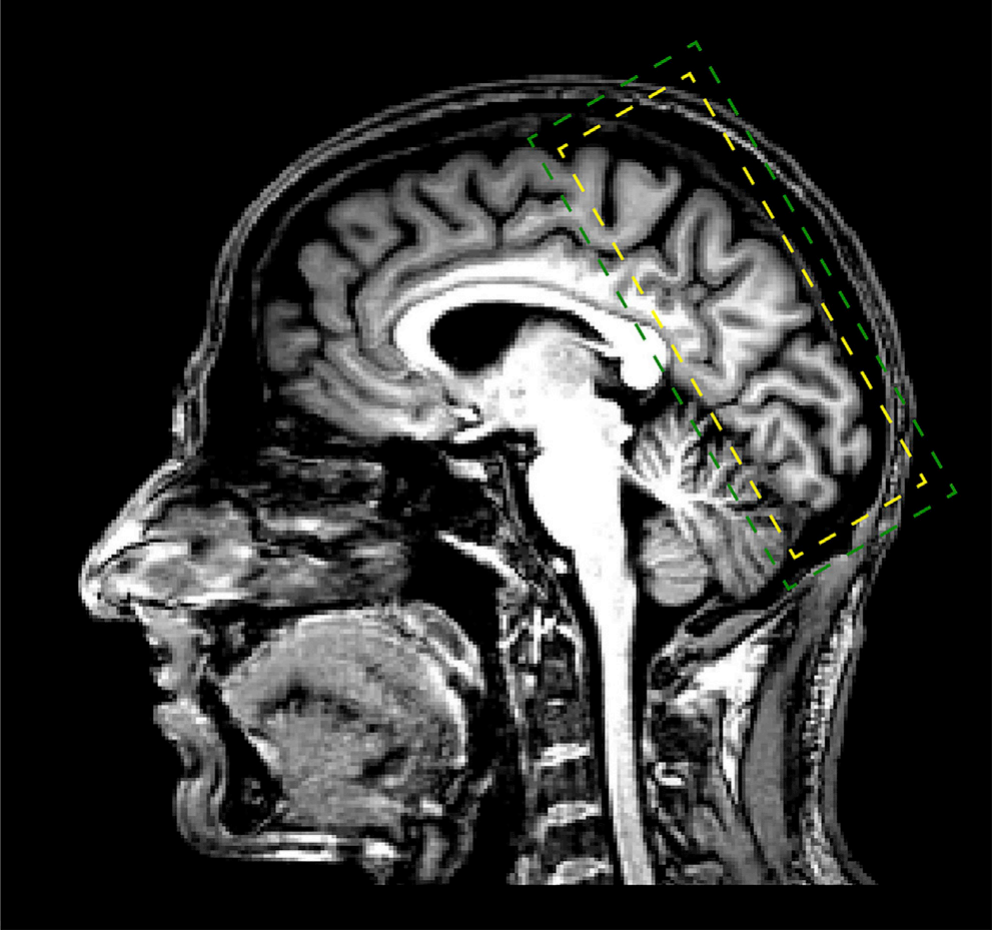
Positioning of the fMRI slab (yellow box) and the shimming volume (green box) Yellow and green boxes show the positioning of the fMRI slab and the shimming volume, respectively.

**Figure 3:**
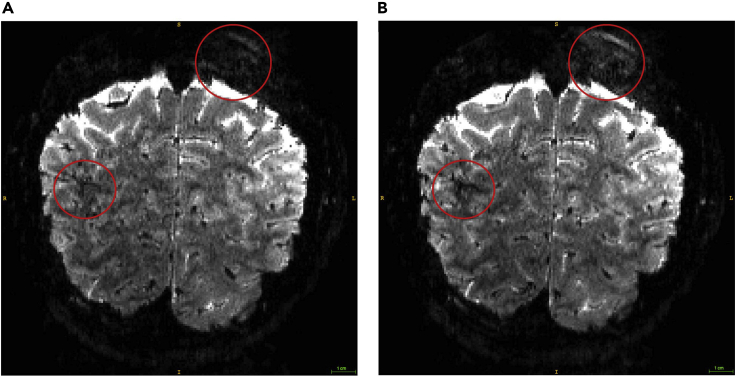
Good vs. poor shimmed functional images (A) and (B) show examples of functional images (single volume, 0.8 mm isotropic) of good vs. poor shimming, respectively. The red circles indicate areas that differ in data quality due to shimming. Poor shimmed image (B) shows more ghosting in upper circles and more signal drop-outs in lower circles comparing to the good shimmed image (A).

1. Register the participant according to the local procedure.
2. Localizer. A scan is run at isocentre of the magnet in order to have a basic localization of the brain with respect to the scanner coordinates.
3. B1 mapping. The B1 map is acquired covering the whole brain and aligned to AC-PC. This will generate a filp angle map. To ensure the correct flip angle is achieved in the target area, load the flip angle map into the 3D tab of the Syngo software (Siemens Terra, VE system). Use the circle ROI tool (tools menu) to draw a ROI in the target area on sagittal image and estimate the following: (900 × Transmit Reference Amplitude) / Mean average. This figure should not be over 300 V. If it is higher than 300 V, use 300 V).
4. Acquire anatomical images using MP2RAGE T1-weighted sequence (TR = 5000 ms, TE = 2.56 ms, FOV = 208 × 208 mm^2^, resolution 0.65 × 0.65 × 0.65 mm^3^, number of slices: 240, slice orientation: sagittal, see the MP2RAGE T1-weighted sequence in the key resources table).
5. Position the fMRI slab on the area of interest (2D Gradient Echo, Echo Planar Imaging (GE-EPI) sequence: TR = 2060 ms, TE = 26.4 ms, FOV = 148 × 148 mm^2^, flip angle: 70°, resolution 0.8 × 0.8 × 0.8 mm^3^, number of slices: 56, partial Fourier = 6/8, GRAPPA factor = 3, Multi-Band factor = 2, bandwidth = 1034 Hz/Pixel, echo spacing = 1.09 ms, see the EPI sequence in the key resources table). At this point the researcher will need to:
  a. Make sure that the area of interest is well contained within the acquisition slab;
  b. Check the phase encoding direction;
  c. Slide through the slices to make sure that the area of interest is fully covered by the slab;
  d. Make sure that there is no wrap-around, ghosting or dropouts in the images.
6. Run one volume of the above scan to check the position of the field of view (FOV).
7. If position is poor, reiterate steps 5 and 6 until good slab coverage has been achieved.
8. Adjust the shim volume (i.e., green box in Figure 2) of the fMRI slab to cover the whole imaging volume (i.e., yellow box in Figure 2). 10 mm were added in all directions of the shim volume to ensure stable numeric optimization and reduce sensitivity to head motion. For imaging at 7T (Siemens Terra scanner), perform B0 shimming as bellow:
  a. Select Options > Adjustments > Transmitter, in ‘amplitude temp’, type the value obtained from B1 mapping. Click on Apply.
  b. Manual Shimming (Siemens Terra, VE line):
    i. Click on Options>Adjustments. Navigate to the ‘Frequency’ tab. Click Go until convergence is reached. Click Apply.
    ii. Repeat this procedure 3 times: Navigate to the 3D Shim tab: measure, calculate and apply.
    iii. Navigate to the ‘Inter. Shim’ tab. Adjust the Z, Y, X, Z^2^ shim currents as directed, trying to decrease the FWHM of the water spectrum. This value should be as close to 30 Hz as possible (see Figure 3 for good vs. poor shimming examples). Click Stop, then Apply.

**CRITICAL:** The researcher will need to ensure that the curves resulting from shimming resemble a Gaussian distribution. If the shimming value is not sufficiently low, the participant may need to be repositioned to ensure that the coil receives the signal properly.

9. Measure the B0 map.
  a. Copy adjustment volume and centre of slices from the fMRI slab (step 5).
  b. Move slices to obtain full brain coverage and run.
10. Acquire an inverted phase encoding direction fMRI scan (e.g., FH: phase encoding direction of functional data from foot to head) used to correct for distortions (5 volumes):
  a. Copy adjustment volume from the B0 map.
  b. Copy Centre of slices with phase encoding direction selected from the fMRI slab (step 5).
  c. Change the phase encoding direction to FH and the number of measurements to 5.
11. Acquire the experimental data.
  a. Copy adjustment volume from the B0 map.
  b. Copy Centre of slices with phase encoding direction selected from the fMRI slab (step 5).
  c. Change the phase encoding direction to HF and the number of measurements based on the experimental design.
12. Run steps 10 and 11 as a pair to reduce the between-run head movement.

**CRITICAL:** At this point the researcher will need to check the data quality. Please ensure that there is no ghosting or signal drop-outs in the region of interest (ROI).

13. Repeat steps 10–12 for all the experimental runs.

**CRITICAL:** At this point the researcher will need to ask the participant to stay as still as possible. Head movement may cause image distortions that cannot be corrected.

14. Save all the data acquired.

## Expected outcomes

**Figure 4:**
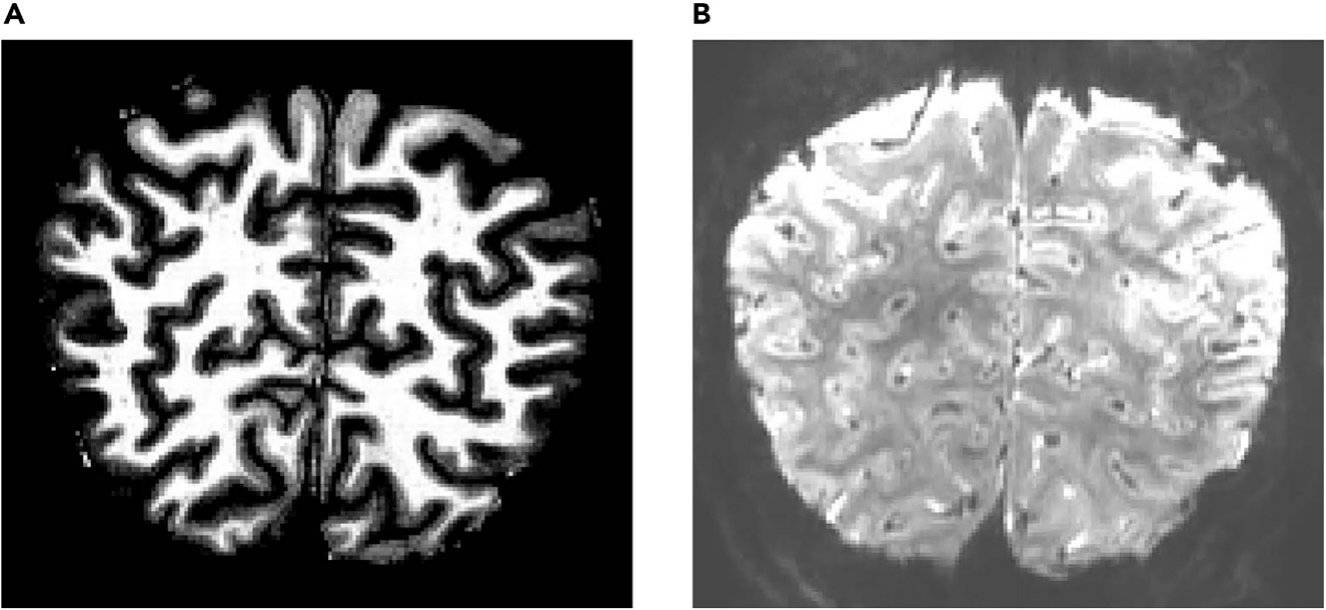
Expected outcomes (A) and (B) show the examples of the anatomical and functional images, respectively.

At the end of the process, you will have collected anatomical (Figure 4A, 50 MB per participant) and functional (Figure 4B, the size of the EPI data is ~2 MB per volume) data. For our experiments, we typically collect 10 runs (~250 volumes per run, that is ~5 GB per participant) brain images at high resolution. The anatomical scans (acquired using MP2RAGE T1-weighted sequence) are used to define the cortical layers, while the functional scans (acquired using GE-EPI sequence) are used to interrogate brain activity underlying cognitive processes (e.g., perceptual learning, adaptation) at a finer scale.

## Quantification and statistical analysis

**Figure 5:**
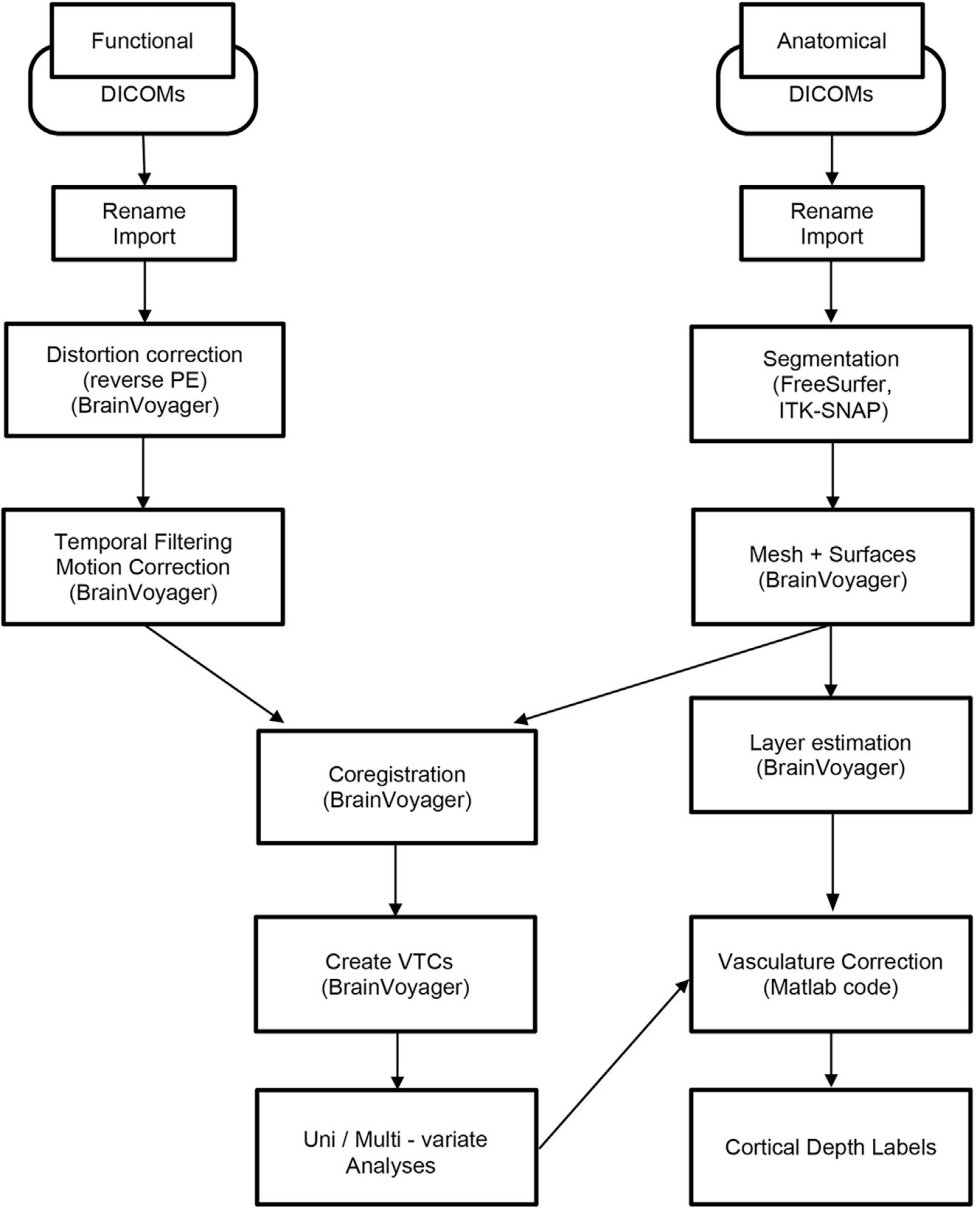
Pipeline overview for analysis of laminar fMRI data

We used a pipeline that combines tools from SPM, FreeSurfer, ITK-SNAP and BrainVoyager (troubleshooting 1, also see Table 1 for the function of each file type) to preprocess the brain imaging data and to correct for vasculature-related confounds. Figure 5 provides an overview and flowchart of all analyses.

**Table 1:** Function of each BrainVoyager file type

BrainVoyager file types	Function of each file type
Volumetric Magnetic Resonance (VMR)	anatomical volume: a 3D file contains the intensity of each voxel.
Functional Magnetic Resonance (FMR)	Text file containing information about functional slices; must be partnered with STC file.
Slice Time Course (STC)	Functional time series organized as 2D slice arrays over time.
Volumetric Time Course (VTC)	Functional time series data organized by 3D volumes over time
Volumes of Interests (VOI)	Volume of interest: coordinates of voxels according to a functional localizer
Volume Map (VMP)	Statistical overlay map

### Preprocessing of anatomical data: Segmentation using FreeSurfer and SPM

**Timing: Approx. 8**–**20 h per participant for a computer (64 bit, 8 cores, i7-6700, 32 GB of memory).**

***Note:*** Disk space: approx. 700 MB per participant

**Figure 6:**
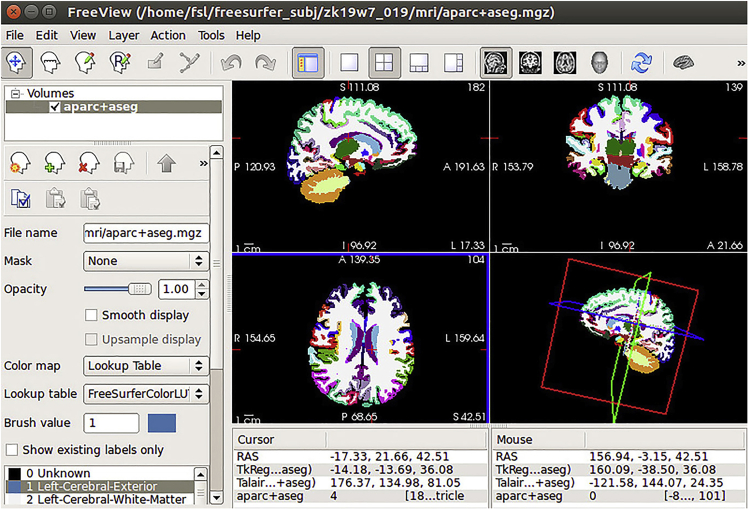
FreeSurfer interface

White matter-gray matter (WM-GM) segmentation is achieved using FreeSurfer (Figure 6, troubleshooting 2) and following the steps described below.

1. Convert DICOM files to Nifti file:
  a. From Matlab, launch SPM toolbox, click on DICOM Import, and select the DICOM files and output directory, click GO.
  b. From a terminal window, set FreeSurfer environment:
    i. Export FREESURFER_HOME=/Your Path to FreeSurfer
    ii. Source $FREESURFER_HOME/SetUpFreeSurfer.sh
    iii. Export SUBJECT_DIR=/Your Path to FreeSurfer/Your folder name
  c. Now run recon-all to obtain surfaces and segmentations:
    i. In the expert.opt file, please set the mris_inflate to 100.
    ii. recon-all –all –s OUTPUTFOLDERNAME –hires –i INPUTFILE.nii –expert expert.opt
  d. Check results in freeview
    i. CD into the {Subject folder} and on the terminal run freeview –v OUTPUTFOLDERNAME/mri/T1.mgz (or brainmask.mgz / wm.mgz)
  e. Convert the results into Nifti format for adjustments in the following steps. From the terminal (while in the OUTPUTFOLDERNAME/mri/ folder):
    i. mri_label2vol –seg ribbon.mgz –temp rawavg.mgz –o ribbon-in-rawavg.mgz –regheader ribbon.mgz
    ii. mri_convert ribbon-in-rawavg.mgz ribbon-in-rawavg.nii
    iii. mri_convert rawavg.mgz rawavg.nii

**CRITICAL:** To save time, the researcher can run these steps in 4–8 participants in parallel.

### Preprocessing of anatomical data: Manual segmentation using ITK-SNAP

**Timing: Approx. 8 h per participant.**

***Note:*** Disk space: approx. 50 MB per participant

WM-GM segmentation can be manually modified for improved results within the region of interest.

2. Assign a name, an integer value and a color to each label. It is possible to save the labels and re-load them in the next session.
3. Labeling Options
  a. Foreground label: the label that will be applied by the tool you use (paintbrush or polygon)
  b. Background label: the label that the foreground will be applied to (if you have foreground label 1 and background label 2, you will DRAW in label 1, but you will only draw over label 2). This prevents you from assigning the same voxel to multiple labels.
4. Select Paintbrush tool (red circle in Figure 7)
  a. Select the label you want to modify.
  b. Select the brush size and style.
  c. Draw voxel by voxel. Draw using the left button on the mouse, and erase with the right button of the mouse.
  d. To toggle the segmentation click S; to change the transparency click A; to change the opacity, click D.

**Figure 7:**
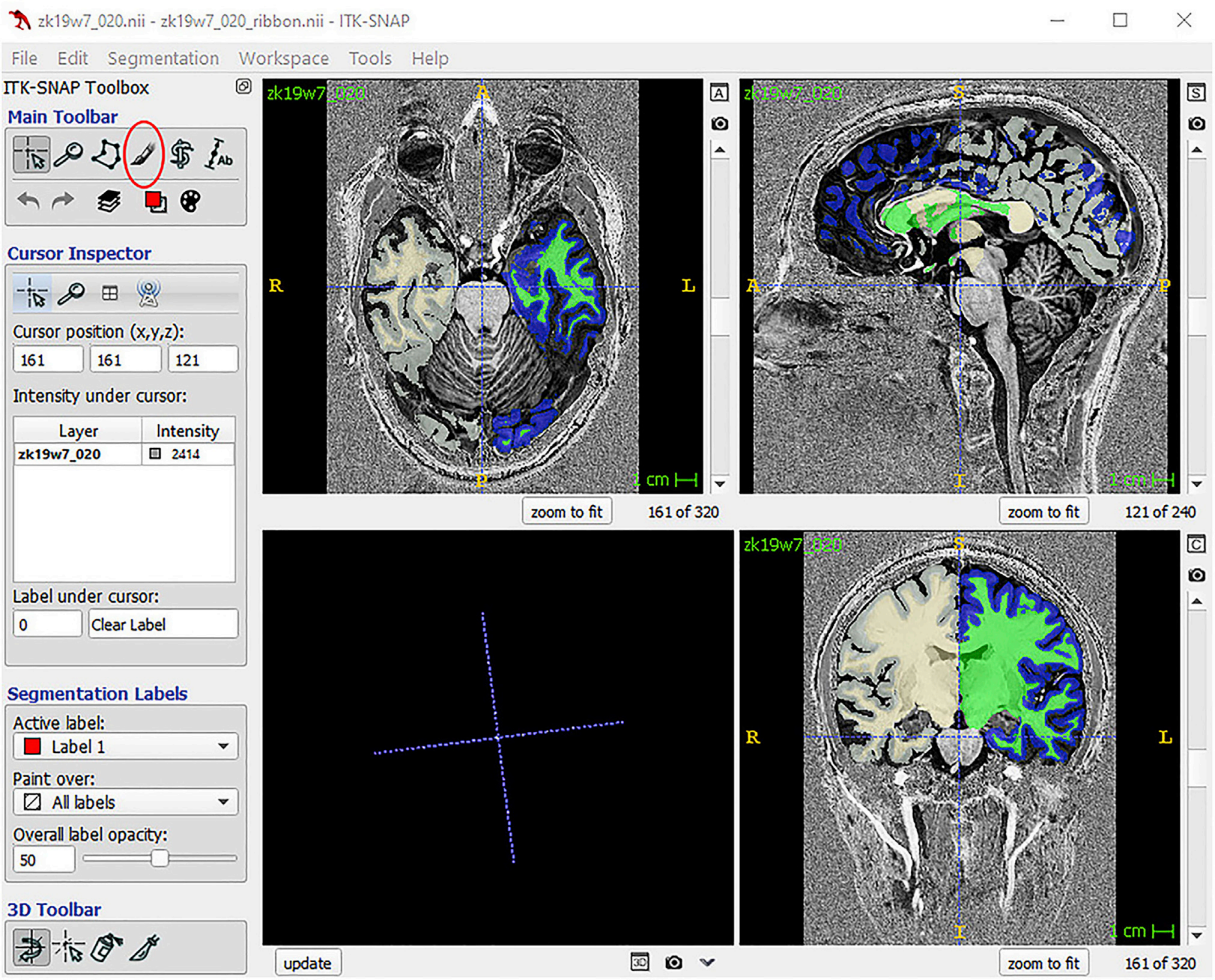
ITK-SNAP interface
5. To assess the QUALITY of the segmentation:
  a. Inspect it in 3D: open the 3D rendering window (bottom left quadrant of the visualization window) and click UPDATE. This will display the segmentation in 3D. Rotating allows you to inspect the whole segmentation.
  b. Check the boundaries between the structures: if some are shallower than others, it is likely that the boundary is shifting from one slice to the other in the 2D viewer (i.e., it has not been defined consistently across slices).
6. Save the resulting scanID_ribbon_fixed.nii.

### Preprocessing of anatomical data: Mesh generation and inflated surfaces using BrainVoyager

**Timing: Approx. 30 min per participant.**

***Note:*** Disk space: approx. 500 MB per participant

To generate cortical surfaces, WM-GM segmentation needs to be completed for each hemisphere: left hemisphere (LH), right hemisphere (RH).

7. In BrainVoyager (Figure 8)
  a. Load the output from ITK-SNAP (Nifti file, scanID_ribbon_fixed.nii) using File/Open Nifti/
  b. Save the resulting segmentation VMR.

**Figure 8:**
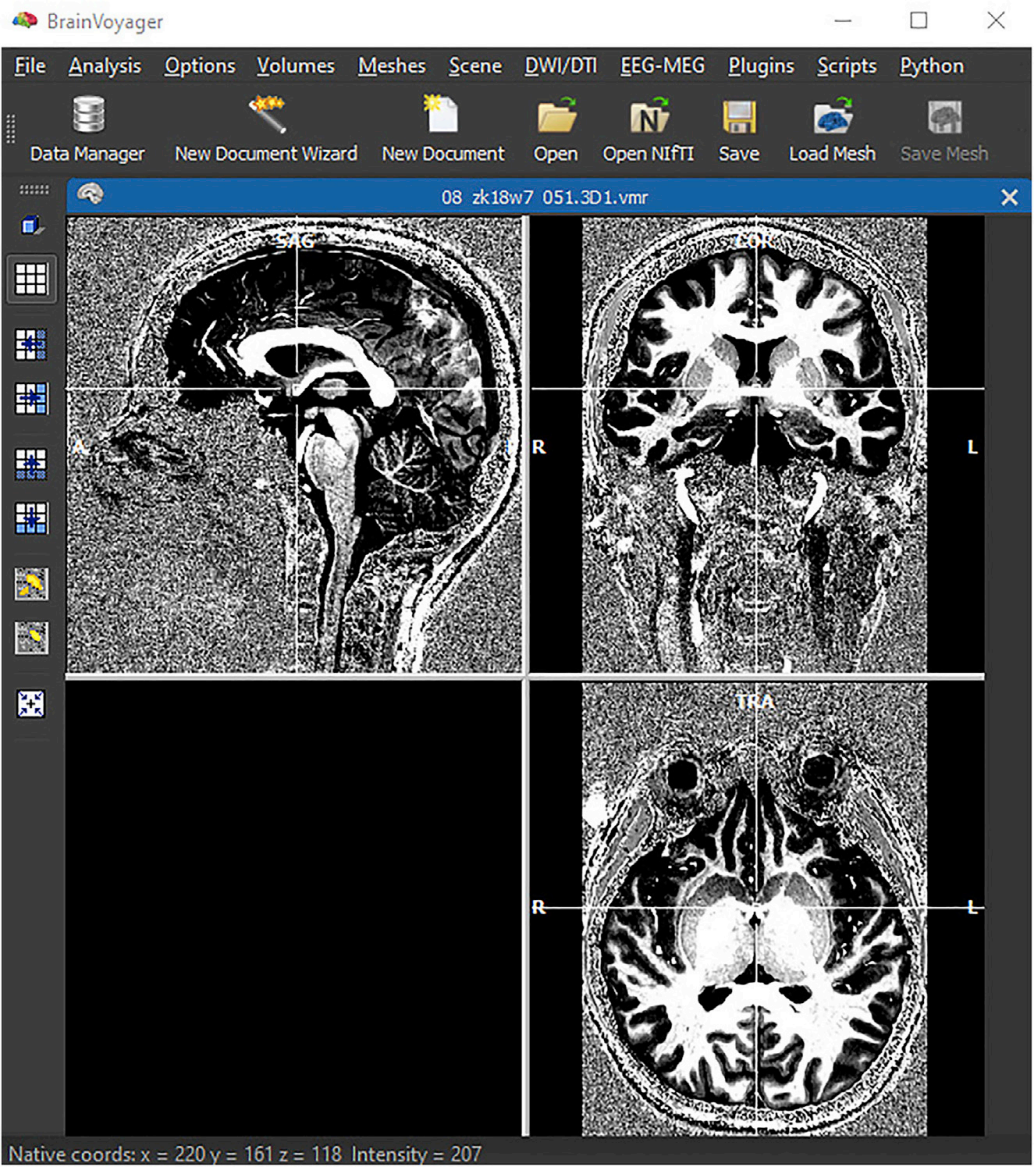
BrainVoyager interface
8. Obtain surfaces for each hemisphere separately:
  a. Save a copy of the resulting segmentation VMR as scanID_segmentation_ LH.vmr
  b. Modify the intensity values using the 3D Volume Tools / Segmentation tab.
    i. Convert intensity values for GM of the left hemisphere (LH) to 100 and for WM of LH to 150.
    ii. Convert intensity values for GM and WM of the right hemisphere (RH) to 0.
  c. Check only GM/WM of LH is selected. To do so, you may load a secondary VMR (File/Load Secondary VMR) and toggle between the segmentation and the second VMR using F8 or F9. If the segmentation is poor, you can also manually adjust it. Set the New value range to 0 and Enable the Draw with mouse option. Using the mouse, delete the part that is not necessary.
  d. Uncheck Show option in the Bounding Box section and select the WM only:
    i. Set Value Range: Min = 0, Max = 149, New = 0, Range.
    ii. Set Value Range: Min = 150, Max = 150, New = 240, Range.
  e. Save the resulting file as scanID_segmentation_LH_WM_mask.
  f. On 3D Volume Tools / Segmentation, click Prep and Reco to generate the mesh.
  g. From Meshes menu, do Advanced Mesh Smoothing (make sure the No Shrinking option is ticked).
  h. From Meshes/Background and Curvature Color, click Curvature in the Calculate Curvature section, then click Smooth, and OK.
  i. Save the result as scanID_segmentation _LH_curvature_smoothed (Figure 9A).

**Figure 9:**
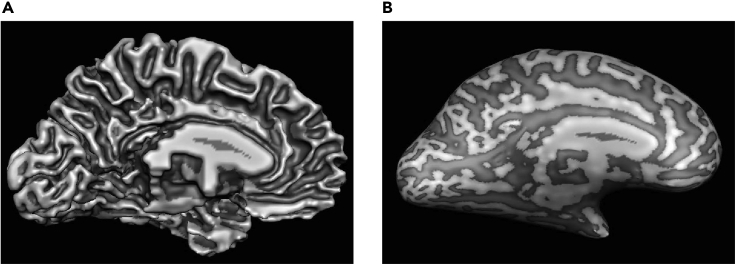
Mesh generation and inflated surfaces in BrainVoyager (A) and (B) show the mesh before and after the inflation.
  j. Inflate the mesh: Meshes/Mesh Morphing
    i. Make sure that the Reference to vertices, colors, edges of secondary mesh corresponds to the mesh you have created (scanID_segmentation_LH_curvature_smoothed or the _RECOSM if available)
    ii. Check Inflation, Update Color, Iterations: 1000, click GO.
  k. Save as scanID_curvature_smoothed_inflated (Figure 9B).
  l. Repeat steps a to k for the right hemisphere.

### Preprocessing of anatomical data: Generate cortical depth layers using BrainVoyager

**Timing: Approx. 1 h per participant.**

***Note:*** Disk space: approx. 500 MB per participant

**Figure 10:**
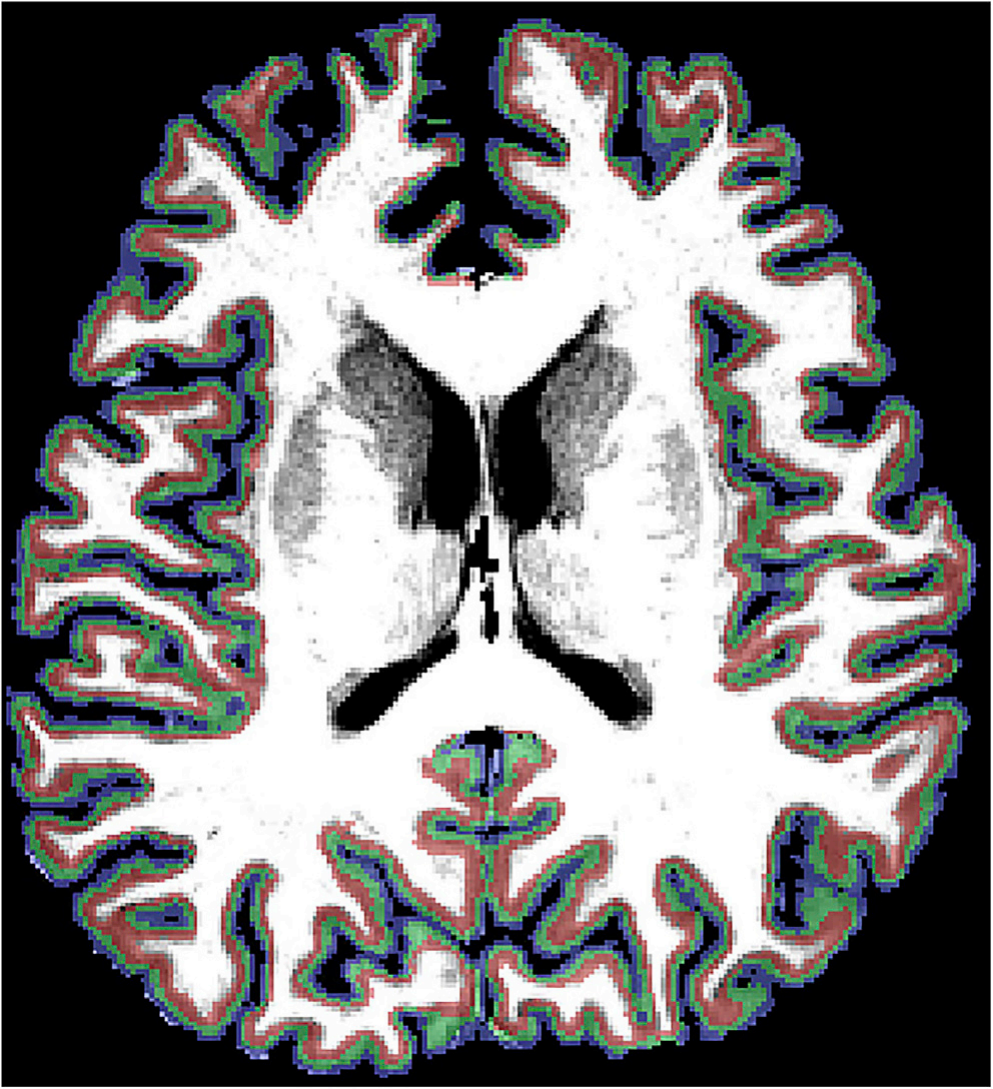
Cortical layers in the anatomical space Layers overlaid on an anatomical image (red, deeper layers; green, middle layers; blue, superficial layers).
    ii. Save the 3 layers as wholeBrain_layers.voi (in the scanID_VMR folder)

9. Load the resulting segmentation VMR [7.b] and do
  a. Volumes/Cortical Thickness Measurement, click GO.
    i. This generates scanID_segmentation_Thickness.vmp
  b. Move to the Mid-GM Volume Tab, click Create Volume and Create VOI
    i. This generates scanID_segmentation_Mid-GM.vmr
10. Having the scanID_segmentation_Mid-GM.vmr loaded
  a. Under 3D Tools/Segmentation tab, click Prep and Reco.
    i. Generate the mesh, do Advanced Smoothing and save it as CorticalDepth_smoothed in VMR folder
  b. Load the scanID_segmentation.vmr
    i. Layers will be projected onto here
    ii. Re-load the mesh generated at point [10.a.i]
  c. Meshes/Cortical Depth Sampling:
    i. Choose the scanID_Thickness file generated in point [9.a.i]
    ii. Click on Save Meshes and fill layers as VOIs, define 4 meshes as default and click GO.
  d. On the scanID_segmentation.vmr file
    i. 3 layers will be displayed (with the Volume-Of-Interest Analysis window open, Figure 10, troubleshooting 3)

### Preprocessing of functional data: Data quality check using BrainVoyager and mcheck toolbox

**Timing: Approx. 30 min per participant.**

Assess data quality by loading FMRs for each run and visually inspect the images, checking for:

11. Head motion: select Options/Time Course Movie. This generates a movie of the timeseries that is displayed in a loop. Check for large jumps in the images throughout the run that indicate head movement and make a note of the volumes where this occurs.
12. Signal: if you are interested in a specific ROI, you can inspect signal strength and pattern by selecting an ROI around the area you are interested in on one of the slices. The timecourse of the selected area will be displayed. Check for artifacts (e.g., signal drifts, drop outs, spikes).
13. Distortions: to visually assess extent of distortions: load the FMR obtained during the inverse phase encoding scans and toggle between the EPI and IPE images.

**Figure 11:**
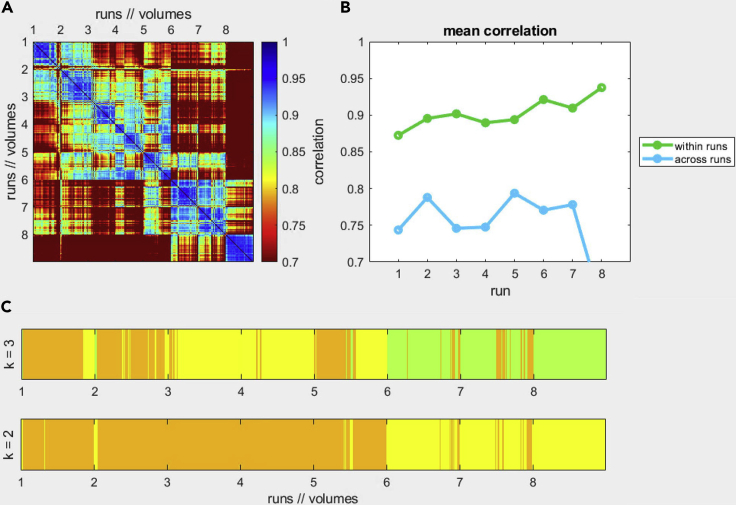
Example output of the mcheck toolbox (A) and (B) show poor correlation across runs and high correlation within runs. We would expect these values to be within the 0.9–1 interval. (C) represents the results of a k-means clustering algorithm: this works on single volumes rather than whole runs and indicates volumes that are more alike based on image properties.

Another tool for assessing data quality is the mcheck toolbox. This is a Matlab toolbox that loads the FMR files and evaluates similarity in time-series by computing correlations of image intensity values (Figure 11).

### Preprocessing of functional data: Distortion correction using COPE

**Timing: Approx. 5 h per participant for a computer (64 bit, 8 cores, i7-6700, 32 GB of memory).**

***Note:*** Disk space: approx. 25 GB per participant

Ensure that the COPE Plugin is installed in BrainVoyager (see https://support.brainvoyager.com/brainvoyager/available-tools/86-available-plugins/62-epi-distortion-correction-cope-plugin for more information).

14. Use the Single Band Reference image (SBRef) to estimate the distortion maps:
  a. For each run, select the SBRef for the IPE and EPI, and estimate the voxel displacement map (VDM).
  b. Once the VDM is estimated, move to Undistort Data (Apply VDM) tab, select the SBRef and EPI images (MultiBand) of the relative run and Apply VDM.
  c. To check the result of the distortion correction, load the scanID_undist.fmr files (one for IPE and one for EPI) and toggle between these. The distortions should be attenuated and the images now should look more alike. (Troubleshooting 4)

### Preprocessing of functional data: Slice scan time correction, temporal filtering, and 3D motion correction using BrainVoyager

**Timing: Approx. 5 h per participant for a computer (64 bit, 8 cores, i7-6700, 32 GB of memory).**

***Note:*** Disk space: approx. 25 GB per participant

**Figure 12:**
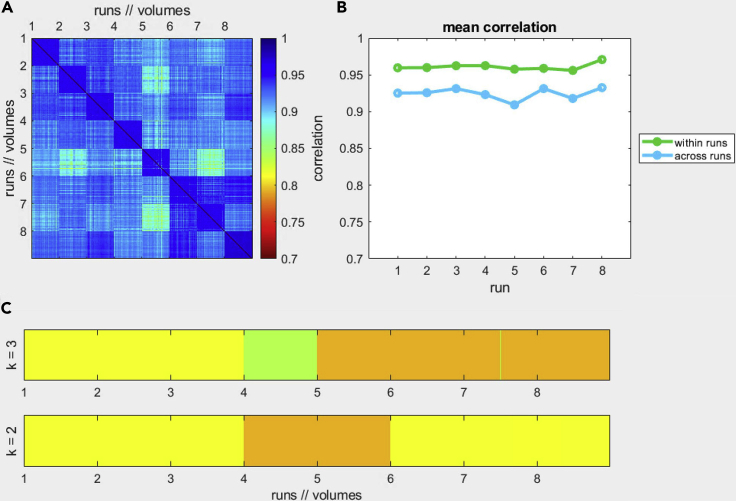
Example output of the mcheck toolbox after motion correction After motion correction, the mean spatial correction across volumes and runs are higher than 0.9.
  d. For each run, load the preprocessed fmr data. Click Options / Convert FMR/DMR to Identical VTC/VDW.

15. For each run:
  a. Load the undistorted FMR data (MultiBand – EPI only) and start the preprocessing pipeline: Analysis / FMR Data Preprocessing.
  b. Use the default options, then select Advanced and on the 3D Motion Options select Use other FMR for intra-session alignment: choose the Single Band Reference of the first EPI run, click OK, and GO.
  c. Check the results of motion correction. BrainVoyager will output the result of the 3D motion correction for each run. This step aligns each acquired volume to the *first* volume of the first run, and this is in turn aligned to the Single Band reference of the first run. To assess the result of the motion correction, run the checkMotion.m function of the mcheck toolbox with the output files from the steps above. The result should present improved mean correlation (see Figure 12 for an example).

**CRITICAL:** Slice scan time correction may be omitted if the experiments use block design. The order of distortion correction and motion correction may be interchanged depending on the data quality for each participant.

### Preprocessing of functional data: Coregistration between the functional data and the anatomical data using BrainVoyager

**Timing: Approx. 30 min per participant for a computer (64 bit, 8 cores, i7-6700, 32 GB of memory).**

***Note:*** Disk space: approx. 50 MB per participant

This process can be divided into two main steps: initial alignment and fine-tuning adjustment. The former is performed using the 3D Volume Tools / Coregistration process (FMR-VMR alignment). The fine-tuning adjustment is performed using Boundary Based Registration (BBR). Note that in order to run BBR, you first need to generate the cortex mesh for the whole brain from the fixed segmentation.

16. Initial Alignment
  Here, a volume (VMR) representation of the respective run of the functional data needs to be created in a space that is close to the native space VMR. This is achieved by running the header-based initial alignment (IA) routine. Besides creating the IA transformation matrix, the header-based step creates and saves a functional volume that is in space close enough to the native space VMR so that the BBR routine is able to find a fine-tuning adjustment (FA) transformation that optimally aligns the two data sets. Here we:
  a. Run standard header-based initial alignement (IA) from the FMR-VMR Coregistration dialog (3D Volume Tools / Coregistration)
  b. Select the SBRef of the first run.
  c. Ensure that Save VMR for BBR is checked in the Initial Alignment tab of the FMR-VMR Coregistration dialog
  d. As output, the IA will generate and save in the same folder of the SBRef a VMR: scanID_#_undist_SCCTBL_3DMCTS_THPGLMFc2-TO-scanID_final_IA_For-BBR.vmr

17. Generate the Cortex Mesh for BBR
  BBR uses a cortex mesh (SRF) as input which is used to align the respective anatomical data (VMR) with another data set. To obtain a suitable cortex mesh, the underlying anatomical data set (VMR) needs to be segmented. Since the FMR data are aligned usually to the intra-session anatomical data set, this VMR file can be used for the segmentation process. It is also possible to first transform the intra-session VMR into ACPC (or TAL) space, perform standard (advanced) segmentation and afterward transforming the segmented brain back into native space. Here, we compute the segmentation in the native space:
  a. After having manually adjusted the WM/GM segmentation using ITK-SNAP and saved the file as scanID_segmentation.vmr, load this in BV
  b. Generate a mask for the WM only and its corresponding Mesh
  c. From 3D Volume Tools/Segmentation, set Value Range: Min = 0, Max = 149, New = 0, Range; set Value Range: Min = 150, Max = 150, New = 240, Range; do Gaussian filtering (to reduce noise), then set Value Range: Min = 0, Max = 0, New = 0, Range; Min = 100, Max = 225, New = 240, range;
  d. Save the mask as scanID_segmentation_WM_mask and then click Prep and Reco
  e. Once the Mesh is generated,
    i. Click Meshes/Advanced Smoothing, check no shrinking and GO.
    ii. Click Meshes/Background and Curvature Color/Calculate curvature, click curvature, then smooth, and save mesh as scanID_segmentation_forBBR_smoothed_curvature.
  f. Use this mesh for the BBR described below.

18. Fine-Tuning Adjustment using Boundary Based Registration (troubleshooting 5)
  BBR will use the cortex mesh in native VMR space that will be aligned to the created functional volume VMR:
  a. Load the created functional data that should be in the same space as the anatomical (mesh) data set (found in the SBRef folder)
  b. Open the created cortex mesh in native space from the functional volume
  c. Open the BBR dialog (Meshes/Boundary-Based Registration), click GO to start BBR
  d. This will produce the spatial transformation FA TRF matrix file.

### Transform the ROIs from the anatomical space to the functional space

**Timing: Approx. 30 min per participant for a computer (64 bit, 8 cores, i7-6700, 32 GB of memory).**

***Note:*** Disk space: approx. 1 MB per participant (depending on the number of ROIs)

19. Transform the anatomical images back to the functional space;
  a. Open the vmr file,
  b. Click Options / Transform Native VMR to FMR-VTC Space.
  c. Select the IA, FA, and FMR files generates in steps 16 and 18, Click Go.
  d. This will generate an anatomical file in the functional space and a transformation matrix that can be used to transform the ROIs.
20. Transform the ROIs back to the functional space;
  a. Open the vmr file in the anatomical space,
  b. Load the ROI that was defined in the anatomical space.
  c. Click Options, and change to the Transformations Tab, select the files generates in point [19.d], Apply TRF.
  d. This will generate the ROIs files in the functional space.

### Correct for superficial bias

It has been shown that the BOLD signal measured using GE-EPI (i.e., T2∗ weighted) is confounded by macro- and micro-vasculature signals (Uǧurbil, et al., 2003; Uludaǧ et al., 2009; Yacoub et al., 2005). The macro-vasculature contribution is due to veins penetrating the gray matter and running through its thickness, as well as large pial veins situated along the surface of the gray matter (Duvernoy et al., 1981). This results in increased sensitivity (i.e., strong BOLD effect) but decreased spatial specificity of the measured signal. The latter can be understood by the mechanics of the draining veins carrying deoxygenated hemoglobin downstream from the true neuronal site of neural activation, leading to a response spatially biased towards the pial surface, an effect known as superficial bias.

Below we describe our approach in controlling for this superficial bias when acquiring data at high resolution using GE-EPI:

21. Exclude the vein voxels.
  a. For each voxel in the region of interest, compute the temporal signal to noise ratio (tSNR). We use tSNR to identify voxels near large veins that are expected to have large variance and low intensity signal due to the local concentration of deoxygenated hemoglobin resulting in a short T2∗ decay time (i.e., dark intensity in a T2∗ weighted image). We identify voxels with low tSNR and check their correspondence with voxels of lower intensities on the T2∗ weighted images.
  b. For each voxel in the region of interest, perform a GLM (stimulus vs. fixation condition) and compute a t score. It has been shown that high t-values on a fMRI statistical map are likely to arise from large pial veins (Kashyap et al., 2018; Polimeni et al., 2010).
  c. Exclude voxels with low tSNR values or t-score values above the 90^th^ percentile of the t-score distribution obtained by the GLM described above from further analysis.
22. Spatial regression analysis (Kok et al., 2016; Koster et al., 2018)
  a. For each voxel in the superficial layers, find the nearest neighbor in the middle layer.
  b. Regress out the mean time course of these voxels assigned to middle layers from the time course of voxels assigned to superficial layers.
  c. Save the time course after the regression for further analyses.

## Limitations

In this protocol, we detail a step-by-step method for using GE-EPI to measure the laminar brain activity in the human cortex. It is important to note that despite the advances afforded by laminar fMRI, GE-EPI is limited by vascular-related contributions to the BOLD signal at the cortical surface resulting in loss of spatial specificity (Kay et al., 2019). Here, we describe possible controls for these potential confounds. Further, CBV imaging using vascular space occupancy (VASO) (Huber et al., 2019) can be used instead of GE-EPI to enhance the spatial specificity of laminar imaging in the human brain.

## Troubleshooting

### Problem 1: BrainVoyager

In this protocol, we used BrainVoyager for data preprocessing (Quantification and Statistical Analysis). BrainVoyager includes up-to-date algorithms specifically for 7T data analyses (e.g., boundary-based registration, equi-volume depth model). The preprocessed data can be read into Matlab for further analyses. Since BrainVoyager is a commercial software, it may not be available to the general public.

### Potential solution

For each step we used BrainVoyager, we listed some free software that can be used instead (Table 2).

**Table 2:** Alternative software for each preprocessing step using BrainVoyager

Preprocessing steps	Alternative software	Links
Mesh generation and Inflated Surfaces	FreeSurfer	FreeSurfer: http://surfer.nmr.mgh.harvard.edu/
Generate Cortical Depth Layers	LAYNII	LAYNII: https://github.com/layerfMRI/LAYNII
Distortion Correction	AFNI or FSL TOPUP	AFNI: https://afni.nimh.nih.gov/ FSL TOPUP: https://fsl.fmrib.ox.ac.uk/fsl/fslwiki/
Slice Scan Time Correction, Temporal Filtering, and 3D Motion Correction	AFNI, FSL, SPM	AFNI: https://afni.nimh.nih.gov/ FSL: https://fsl.fmrib.ox.ac.uk/fsl/fslwiki SPM: http://www.fil.ion.ucl.ac.uk/spm/
Coregistration between the functional data and the anatomical data	AFNI, FSL, ANTs	AFNI: https://afni.nimh.nih.gov/ FSL: https://fsl.fmrib.ox.ac.uk/fsl/fslwiki ANTs: http://stnava.github.io/ANTs/

### Problem 2: FreeSurfer segmentation

For some participants, FreeSurfer cannot detect the brain automatically and you may encounter a skull strip error (Preprocessing of Anatomical data: Segmentation using FreeSurfer and SPM).

### Potential solution

Use the proton images to generate the brain mask. Use this mask to exclude the noise background, and then run the automatic segmentation again.

### Problem 3: Definition of cortical layers

For some participants, the whole-mesh cortical depth sampling method may lead to suboptimal geometric sampling (Preprocessing of Anatomical data: Generate Cortical Depth Layers using BrainVoyager).

### Potential solution

Try to use the 2D Grids tool of BrainVoyager. Please find more details at https://download.brainvoyager.com/bv/doc/UsersGuide/HighResDataAnalysis/RegularGridCorticalDepthSampling.html. You can also try to use LAYNII instead.

### Problem 4: Distortion correction

For some participants, you may still encounter large residual distortions after running the distortion correction using COPE (Preprocessing of Functional data: Distortion correction using COPE).

### Potential solution

Use AFNI or FSL TOPUP for distortion correction.

### Problem 5: Alignment between functional and anatomical images

For some participants, the alignment between functional and anatomical images is not satisfactory (Preprocessing of Functional data: Coregistration between the functional data and the anatomical data using BrainVoyager).

### Potential solution

Truncate the functional data (i.e., set all the time series of voxels out of the ROI to 0) to improve the coregistration of the ROI. Use recursive BBR (Van Mourik et al., 2019) or Advanced Normalization Tools (ANTs) to run the alignment.

## Resource availability

### Lead contact

Further information and requests for resources should be directed to the lead contact, Zoe Kourtzi (zk240@cam.ac.uk).

### Materials availability

This protocol did not generate any unique reagents.

### Data and code availability

Further information and requests for the raw datasets and code generated by this protocol should be directed to and will be fulfilled by the lead contact, Zoe Kourtzi (zk240@cam.ac.uk).
